# Effect of long-term care insurance policy on depression in non-disabled people: evidence from China

**DOI:** 10.1186/s12889-024-18375-3

**Published:** 2024-04-04

**Authors:** Wenjing Jiang, Hongyan Yang

**Affiliations:** 1 Center for Social Security Studies, Wuhan University, 430072 Wuhan, China; 2 School of Political Science & Public Administration, Wuhan University, 430072 Wuhan, China

**Keywords:** Long-term care insurance, Differences-in-differences, Depression, Non-disabled people

## Abstract

**Background:**

Policy effect might be multidimensional and spill over to non-recipients. It is unclear how the implementation of Long-Term Care Insurance (LTCI) policy affects depression in non-disabled people and how this effect differs in different non-disabled groups.

**Methods:**

Using time-varying differences-in-differences method and nationally representative health survey data in wave 2011, wave 2013, wave 2015 and wave 2018 from the China Health and Retirement Longitudinal Study, we assessed the effect of LTCI policy on depression in non-disabled people aged 45 years and older, and discussed the heterogeneity of effect across different population characteristics: retirement, financial support and social participation status.

**Results:**

We found LTCI policy statistically significant reduced depression by 0.76 units in non-disabled people compared to non-pilot cities. Depression in non-disabled people who unretired, with financial support and without social participation was reduced by 0.8267, 0.7079 and 1.2161 units, respectively.

**Conclusions:**

Depression in non-disabled people was statistically significant reduced because of LTCI policy in China, and non-disabled people who unretired, with financial support and without social participation benefited more from LTCI policy. Our findings highlight the depression-reducing effect of LTCI policy in non-recipients and suggest that non-disabled people who unretired, with financial support and without social participation should be concerned during LTCI policy progress.

## Introduction

### Background

In response to ageing and increasing demand for long-term care (LTC), many countries have established LTC systems, such as France, Italy and Portugal, etc. [1]. To support countries on implementing sustainable and equitable LTC systems, the World Health Organisation (WHO) in 2021 has released a new *framework for countries to achieve an integrated continuum of long-term care* [2]. According to the Organisation for Economic Co-operation and Development (OECD) data from 2022, LTC recipients aged 65 years old in institutions (other than hospitals) reach 0.96 million in Japan and at home reach 1.08 million in Spain [3].

After considering factors such as the degree of aging, the level of economic development and the trial-and-error cost, the Chinese government issued an official document in 2016 requiring 15 pilot cities to implement Long-Term Care Insurance (LTCI) policy. Other cities were not allowed to implement LTCI policy without government permission. The core connotation of LTCI policy was to raise funds in the form of social insurance, and provide funds or service guarantees for long-term disabled people in basic life care and basic medical care. Presently, in pilot cities the combination of multiple financing channels was the mainstream practice, access to cash or services was a staple of benefits, and handling agencies were mainly composed of social medical insurance institutions and commercial insurance institutions.

Depression is a common mental disorder and has become a challenging public health problem. The WHO predicted that 5% of adults worldwide suffer from depression, and in 2019 the WHO’s *Comprehensive Mental Health Action Plan* was approved the extension to 2030 [4]. The consequences on labour market due to depression was egregious, study shown that depression reduced the employment probability among people aged 50–64 by 22–51% points, increased sick leave by 7.2 days per year, and the annual cost to Europe was EUR 118 billion [5]. Depression is influenced by many factors [6]. Previous studies found that young-old adults were associated with more barriers to mental health treatment [7], female was more likely to be depressed when facing loneliness because of declining estrogen levels in their golden age [8], and people with secondary and higher education were more likely to seek professional help for depression than those with no formal education [9]. Appropriate social participation was beneficial to improve depression because of the U-shaped relationship between social participation and depression [10], and frequent social contact was important to depression improvement for older empty nesters with disabilities [11]. Previous studies also found that higher community socioeconomic status was associated with lower individual depressive symptom among middle-aged and older adults [12], depressive risk of the elderly was higher in living alone compared with not living alone in China [13, 14], and regular contact with children was important for the mental health of older adults [15]. Severe depression was associated with poorer social support [16], people with employment were associated with lower depression rates [17], stigma prevents people from seeking mental health help [18].

The main objectives of our study were assessing the effect of LTCI policy on depression in non-disabled people using time-varying difference-in-difference (DID) method and nationally representative health survey data and discussing the heterogeneity of effect across different population characteristics: retirement, financial support, and social participation status. Our contributions were reflected in the following aspects. Firstly, in theoretical contributions, we comprehensively applied Knowledge-Attitude-Practice (KAP) theory, social identity theory, social exchange theory, and activity theory to discuss the relationship between LTCI and depression in a theoretical sense. Secondly, in practical contributions, our study provided ideas for how developing countries can promote healthy ageing in the LTCI policy process. Thirdly, in social contributions, our study proved that the effect of LTCI policy spilled over to non-recipients, which is conducive to the early intervention of depression in non-disabled people. Fourthly compared with the current literature, we look beyond the research perspectives of people with disabilities and caregivers to non-recipients who have been previously overlooked and explored the policy effect of LTCI from the perspective of non-recipients.

### Literature review and hypothesis

Regarding the relationship between LTCI and depression, previous literature focused on system recipients, i.e., the disabled and caregivers. LTC system might positively affect the depression of recipients [19–22]. Previous studies shown that meeting the LTC needs of older adults could reduce their depression [19], the life quality of people with major depression was improved by nursing home care [20], and some services provided by LTC institutions could significantly relieve depression in older adults [21, 22]. Study found that preventive home visiting programs in Japan’s LTCI system significantly improved depressive symptoms in older adults [23].

LTCI system might also affect the depression level in caregivers. Informal caregivers of people with psychological symptoms might experience psychological burden when performing daily nursing duty [24], if recipient had mental condition, caregivers’ depression rate might higher [25]. On the one hand, the effect of LTCI on caregivers’ depression levels might not be positive. The survey results showed that LTCI did not significantly improve the depression of family caregivers, the probability of depression among the elderly in the community who received LTCI services was as high as 34.2% [26]. Although the depression rate of family caregivers (48.4%) decreased slightly after the implementation of LTCI in 2000 compared with 50.3% before LTCI, the odds ratio of depression among spouse caregivers (2.92) increased compared with that before LTCI (1.38) [27]. In addition, Japan’s LTCI reform in 2006 led to a significant increase in depressive symptoms among family caregivers [28]. On the other hand, the effect of LTCI on caregivers' depression levels might also be positive. Study has pointed out that LTCI policy in China significantly reduced the depression level of spouse caregivers, and this effect was more obvious in male, urban and eastern regions [29]. Some scholars found that receiving a family care allowance was beneficial to improve health-related quality of life (including activities of daily living, physical pain, depression, etc.) of family caregivers in Spain [30].

However, policy effects might be multidimensional and spill over to non-recipients. Presently, it is unclear how the implementation of LTC system affects depression in non-disabled people and how this effect differs in different non-disabled groups. LTCI policy practice in China provided evidence to study the health, health inequalities and LTCI policy effect among non-disabled people.

Knowledge-Attitude-Practice (KAP) theory is a behavioral intervention theory, widely used in health education and health promotion fields [31–34]. KAP theory emphasizes that knowledge is the basis of behavior change, attitudes are the driving force of behavior change, people through the acquisition of knowledge and positive thinking, their attitudes will change, and then lead to changes in behavior. This theory explains how knowledge and attitudes affect health behavior, and reveals the logical progressive relationship of them, that is, information-knowledge-attitude-practice-improve health. Previous studies shown that based on the knowledge-belief-practice model, nurse-led health education improved the self-management ability, satisfaction and compliance of elderly patients successfully [32]. Interventions such as training frontline health workers and raising people’s awareness could lead to positive health outcomes -- enabling more people with mental disorders to be diagnosed and managed [35], prediabetes education programs could significantly improve knowledge, attitudes and practices in preventing diabetes [36] and health disorders among solid waste workers [37]. LTCI policy might be an influence factor that changing health behaviors in non-disabled people. During the LTCI policy process, health knowledge such as disability level and disability care was effectively disseminated, which might help non-disabled people form health cognition and healthy behavior, thereby reducing depression. Therefore, based on KAP theory and previous studies, we proposed:

#### Hypothesis 1

LTCI reduced the depression in non-disabled people.

Depression reduction effect on non-disabled people brought by LTCI policy might vary from population characteristics. Social identity theory argues that individuals recognize both group membership and the emotional and valuable significance that comes with group membership, through social categorization, social comparison, and positive distinction individuals establish social identity, and identify with ingroups or discriminate against outgroups on cognition, emotion and behavior [38]. Social identification has vital implications for improving mental and physical health [39], members of lower social classes or ethnic minority groups might suffer more negative health outcomes [12, 40]. This theory provides ideas for explaining depression between retired and non-retired people. Social identity might largely affect a person’s behavior, retirees often lack belonging sense, because their identification with retirement life has not yet been fully established after retired. Previous studies found that retirement might lead to psychological distress, social distancing and depression risk [41], the shrank of social networks size after retirement resulted in an 18.6% increase in depression probability in retired people [42], while people who were employed were associated with lower depression rates [17]. Therefore, based on social identity theory and previous studies, we proposed:

#### Hypothesis 2

The effect of LTCI on depression in non-disabled people varied with retirement.

Social exchange theory asserts that pursuit personal interests is a basic trait of human nature, interaction between people is based on exchange rewards, and resources availability and quantity is the premise of exchange [43]. A reciprocal and balanced relationship would contribute to increase the health and well-being level [44]. The inability for the elderly to invest resources was the main reason for the decline in interaction between the elderly and the young [45]. If parents and children had a poor interaction or reciprocal relationship, conflict could lead to neglect, abuse, or mistreatment. Previous studies found financial support from future generations alleviated older adults’ depressive, for every unit increased in the logarithm of financial support, the predicted CES-D score decreased by 29% [46]. Similarly, financial support from family members/friends could also alleviate depression caused by income loss among young people [47]. Therefore, based on social exchange theory and previous studies, we proposed:

#### Hypothesis 3

The effect of LTCI on depression in non-disabled persons varied with financial support.

Activity theory believes that older adults are more likely to age successfully when they remain active, that is, when they remain socially engaged, accept productive roles in society (e.g., participation in social groups or activities) while substituting roles they lost with ageing [48]. According to this theory, positive aging means preserving the activities and attitudes of midlife as much as possible. Some studies reported that social participation could prevent mental health problems, known as depressive symptoms. Participation in productive activities was associated with fewer depressive symptoms for older adults with dual sensory loss [49]. People who were highly active had lower depressive symptoms [10, 11, 50]. After controlling for the confounding effects of aging, individual demographic differences, and health status, continued or initiated participation in social activities later in life was significantly associated with a reduction in depressive symptoms in older adults [51].Social participation not only regulated depression caused by chronic diseases [52], but also reduced the depression risk caused by air pollution exposure among older adults [53]. Therefore, based on activity theory and previous studies, we proposed:

#### Hypothesis 4

The effect of LTCI on depression in non-disabled persons varied with social participation.

## Methods

### Data sources and study sample

Our data was from the China Health and Retirement Longitudinal Study (CHARLS) in wave 2011, 2013, 2015 and 2018. CHARLS collected a high quality nationally representative sample of Chinese residents aged 45 and over, adopting multi-stage stratified PPS sampling. The baseline national wave was fielded in 2011 including about 10,000 households and 17,500 individuals in 150 counties/districts and 450 villages/resident committees. CHARLS questionnaire included demographics, family structure/transfer, health status and functioning, biomarkers, health care and insurance, work, retirement and pension, income and consumption, assets, and community level information modules.

**Fig. 1:**
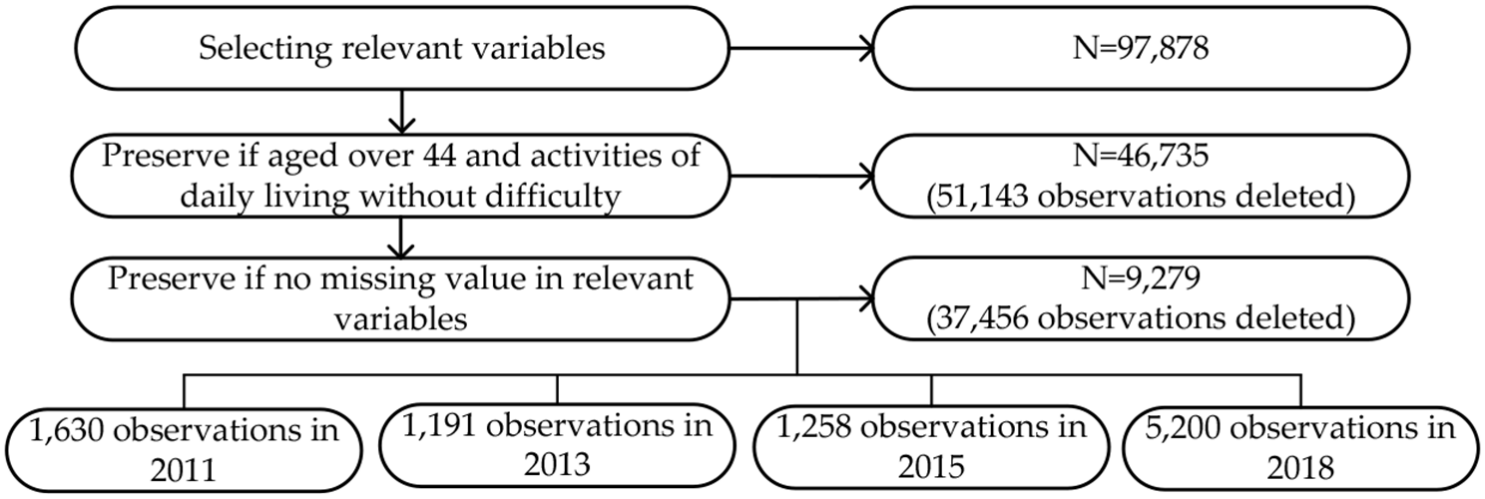
Sample selection process

In sample screening (see Fig. 1), after selecting variables relevant to our study, 97,878 observations were preserved. Then, we preserved the non-disabled people who aged over 44 and had no difficulty in activities of daily living (ADL), 46,735 observations were preserved. Finally, we excluded missing values for relevant variables, 9,279 observations were preserved. The final sample consisted of 1,630 non-disabled people in the 2011 wave, 1,191 in the 2013 wave, 1,258 in the 2015 wave, and 5,200 in the 2018 wave.

**Fig. 2:**
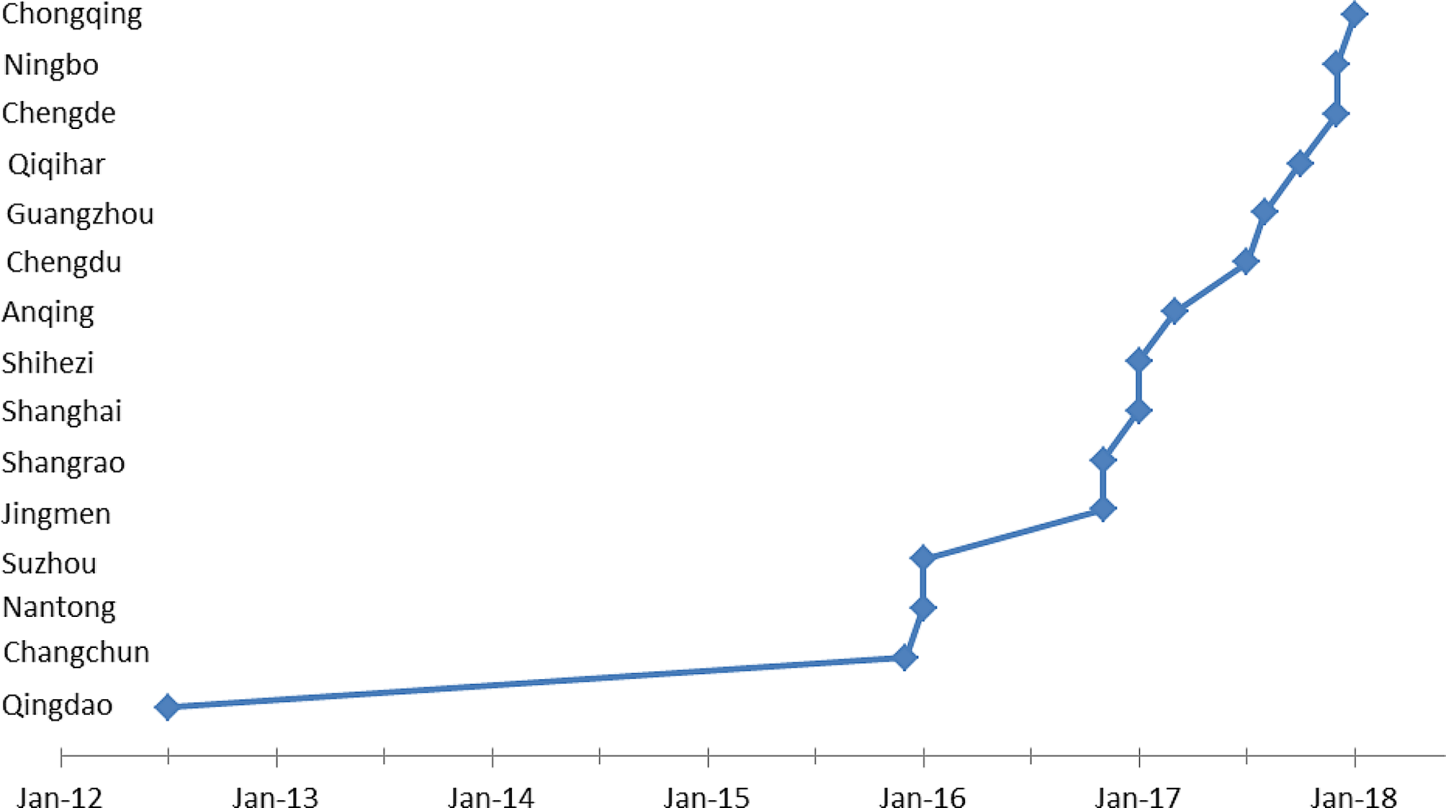
Pilot cities evolution

LTCI data was taken from policy documents in pilot cities. Based on these data, we were able to clarify the pilot cities list and judge whether the city carried out this policy change by the date of the survey. The Chinese government included 15 cities in the first pilot list, 27% of them carried out policy change in 2016, and 60% of them carried out policy change after 2016. Figure 2 reports the pilot cities evolution.

### Measures

#### Dependent variable: depression

The dependent variable was depression. The depression score was taken from 10-item Center for Epidemiologic Studies Depression Scale (CES-D-10) short form. CES-D-10 in CHARLS measured 10 responses to depressive symptoms, including “how often the respondent felt depressed during the past week”, “how often the respondent felt that everything they did was an effort during the past week”, “how often the respondent felt their sleep was restless during the past week”, “how often the respondent felt happy during the past week”, “how often the respondent felt lonely during the past week”, “how often the respondent felt bothered by things that do not usually bother them during the past week”, “how often the respondent felt they could not get going during the past week”, “how often the respondent felt they had trouble keeping their mind on what they were doing during the past week”, “how often the respondent felt hopeful about the future during the past week” and “how often the respondent felt fearful during the past week”. CES-D-10 score ranged from 0 to 30, a higher score meant more severe depression.

#### Independent variable: LTCI

The independent variable was the implementation of LTCI. The results of LTCI ranged from 0 to 1. Value 1 indicated that this city not only belonged to pilot list but also implemented the policy in survey year. Otherwise, the value was 0.

#### Control variables

All analyses included a series of control variables associated with individual depression according to previous studies [42, 46, 47, 52–54]. Among them, the sociodemographic characteristics included age, gender, and education. Lifestyle characteristics included social participation, smoking and physical activity. Family characteristics included the number of cohabited family members, the number of children, household income, living environment, financial support, and emotional support. Social support characteristics included receiving public pension and covering by public health insurance. The variables analyzing heterogeneity were based on retirement differences, financial support differences and social participation differences.

Table 1 reports the descriptive statistical of main variables in 9,279 non-disabled people. The mean depression level in non-disabled people exceeded 7.5. Their average age was about 61.2 years, 47.3% were male. Their average education level was between sishu and elementary School. 49% had social participation last month. 42.4% had smoke ever. 83.3% participated in physical activity weekly. The average number of cohabited family members was about 3 and number of children was 2.7. The logarithmic value of household consumption was about 9.6 per year. 59.7% lived in rural village. 70.4% received financial support from children/grandchildren last year. 90.7% received emotional support from children weekly. 33.7% currently were receiving public pension. 95.2% were covering by public health insurance. 16% completed retirement process.

**Table 1:** Definition and descriptive statistics

Variable	Definition	Mean	Std Dev	Min	Max	N
Depression	CES-D-10 score ranges from 0 to 30	7.5797	5.9118	0	30	9,279
Treat*Post	Belong to pilot city and the policy was implemented in the survey year = 1, otherwise = 0	0.1268	0.3328	0	1	9,279
Age	Age of respondent in the survey year	61.2347	9.4167	45	95	9,279
Gender	Male = 1, female = 0	0.4728	0.4993	0	1	9,279
Education	No formal education illiterate = 1, did not finish primary school but capa = 2, sishu = 3, elementary school = 4, middle school = 5, high school = 6, vocational school = 7, two/three year college/associate degree = 8, four year college/bachelor’s degree = 9, post-graduated (master/ phd) = 10	3.4254	1.8945	1	10	9,279
Social participation	Respondent participated in any social activities last month, yes = 1, no = 0	0.4922	0.5000	0	1	9,279
Smoking	Respondent reports ever smoking, yes = 1, no = 0	0.4244	0.4943	0	1	9,279
Physical activity	Do physical activity at least 10 min weekly, yes = 1, no = 0	0.8336	0.3725	0	1	9,279
Cohabitation arrangement	The number of cohabited family members	2.9703	1.5514	1	14	9,279
Number of children	The number of children	2.6612	1.3327	0	10	9,279
Household income	Logarithmic household consumption last year	9.5879	1.6539	0.6931	17.4806	9,279
Living environment	Lives in rural or urban, rural village = 1, urban community = 0	0.5967	0.4906	0	1	9,279
Financial support	Received any transfer from children/grandchildren last year, yes = 1, no = 0	0.7042	0.4564	0	1	9,279
Emotional support	Contact children in person/phone/email weekly, yes = 1, no = 0	0.9071	0.2903	0	1	9,279
Public pension	Receiving public pension, yes = 1, no = 0	0.3373	0.4728	0	1	9,279
Public health insurance	Covering by public health insurance, yes = 1, no = 0	0.9526	0.2125	0	1	9,279
Retirement	Completing retirement process, yes = 1, no = 0	0.1599	0.3666	0	1	9,279

### Statistical analysis

The DID method is a quasi-experimental technique that measures the causal effect of some nonrandom intervention [55]. Due to the different time of entering the experimental period in each experimental group, we established the time-varying DID method with reference to other scholars [56]:

$$ {DEP}_{ict}={\alpha }_{1}+{\theta }_{1}{{Treat}_{ic}*Post}_{ct}+{\lambda }_{1}{Z}_{ict}+{\eta }_{c}+{\mu }_{t}+{\epsilon }_{ict} \left(1\right)$$

Where $$ {DEP}_{ict}$$ denotes the depression status of non-disabled people *i* who lives in the city *c* in time *t*. $$ {Treat}_{ic}$$ represents the pilot status of non-disabled people *i* who lives in the city *c*. $$ {Post}_{ct}$$ represents the policy post status of city *c* in time *t*. $$ {\theta }_{1}$$ measures the impact of this policy change on depression. $$ {\lambda }_{1}$$ is a vector of control variables $$ {Z}_{ict}$$. $$ {\eta }_{c}$$ and $$ {\mu }_{t}$$ represent city and year fixed effect. $$ {\epsilon }_{ict}$$ represents random perturbations that affect depression. Finally, the standard errors are clustered at city level to correct for possible autocorrelation and heteroscedasticity.

The parallel trend hypothesis is a key prerequisite for constructing the time-varying DID method, which requires that the depression trends of non-disabled people in pilot cities and non-pilot cities must be parallel before policy implementation. Therefore, we used the event research method proposed by other scholars [57]to establish a parallel trend test model:

$$ {DEP}_{ict}={\alpha }_{1}+{\theta }_{t}\sum _{-3}^{6}{{Treat}_{ic}*Post}_{ct}+{\lambda }_{1}{Z}_{ict}+{\eta }_{c}+{\mu }_{t}+{\epsilon }_{ict} \left(2\right)$$

Where ${\theta }_{t}$ reflects the depression disparities in pilot and non-pilot cities in the *t* year of the policy post. The data 4 years before policy was very little, so we aggregated the data 4 years before into year − 4 and considered year − 4 as the base year. Other variables are synonymous with Eq. (1).

## Results

### Main results

Table 2 reports the stepwise regression results obtained using the time-varying DID model. When control variables were not considered, the coefficient of Treat*Post was − 0.7378 (significant levels was 5%). When all control variables were included into model, the coefficient of Treat*Post was − 0.76 (significant levels was 1%), which indicated LTCI policy statistically significant reduced depression by 0.76 units in non-disabled people. Thus, hypothesis 1 was validated.

**Table 2:** The results of the time-varying DID model of non-disabled people

Variables	(1)	(2)	(3)	(4)	(5)
	Depression	Depression	Depression	Depression	Depression
Treat*Post	−0.7378**	−0.7745***	−0.7766***	−0.7557**	−0.7600***
	(0.2932)	(0.2896)	(0.2886)	(0.2893)	(0.2893)
Age		0.0052	0.0035	−0.0019	−0.0021
		(0.0076)	(0.0075)	(0.0084)	(0.0092)
Gender		−1.4006***	−1.8924***	−1.9533***	−1.9509***
		(0.1323)	(0.1704)	(0.1707)	(0.1703)
Education		−0.3258***	−0.2949***	−0.2050***	−0.2034***
		(0.0387)	(0.0381)	(0.0383)	(0.0382)
Social participation			−0.5362***	−0.4336***	−0.4300***
			(0.1278)	(0.1258)	(0.1255)
Smoking			0.6295***	0.5745***	0.5726***
			(0.1897)	(0.1888)	(0.1883)
Physical activity			−0.2781	−0.2336	−0.2313
			(0.1746)	(0.1762)	(0.1764)
Cohabitation arrangement				0.0083	0.0090
				(0.0443)	(0.0443)
Number of children				−0.0320	−0.0329
				(0.0610)	(0.0607)
Household income				−0.4160***	−0.4155***
				(0.0466)	(0.0465)
Living environment				0.4334**	0.4386**
				(0.1997)	(0.2003)
Financial support				−0.2102	−0.2076
				(0.1300)	(0.1301)
Emotional support				−0.6768***	−0.6780***
				(0.2295)	(0.2288)
Public pension					0.0080
					(0.1540)
Public health insurance					−0.2694
					(0.2624)
Fixed city	Yes	Yes	Yes	Yes	Yes
Fixed year	Yes	Yes	Yes	Yes	Yes
_cons	7.6733***	9.1392***	9.5947***	14.1355***	14.3851***
	(0.0372)	(0.4898)	(0.5187)	(0.7965)	(0.8519)
N	9279	9279	9279	9279	9279
R^2^	0.071	0.101	0.105	0.118	0.118

*Note* *, **, and *** represent significant levels of 10%, 5%, and 1%, respectively. The values in parentheses are city-level clustering robust standard errors

### Parallel trend, robustness, and placebo tests

Table 3 reports the parallel trend test results at 95% confidence interval with considering year − 4 as the base year. The estimate values were not statistically significant in year − 2, year − 1 and year 0, while after year 0 they were statistically significant and their sign direction consistently coincided with main result. Therefore, the sample generally passed the parallel trend test.

**Table 3:** Parallel trend test

Variables	Depression
Treat*Post year − 3	0.948*
	(0.546)
Treat*Post year − 2	−0.246
	(0.507)
Treat*Post year − 1	−0.098
	(0.589)
Treat*Post year 0	0.286
	(0.373)
Treat*Post year 1	−1.031**
	(0.397)
Treat*Post year 2	−1.351*
	(0.802)
Treat*Post year 3	−2.010***
	(0.507)
Treat*Post year 6	−3.635***
	(0.435)
Control variables	Yes
Fixed city	Yes
Fixed year	Yes
_cons	14.481***
	(0.857)
N	9279
R^2^	0.119

*Note* *, **, and *** represent significant levels of 10%, 5%, and 1%, respectively. The values in parentheses are city−level clustering robust standard errors

We tested the robustness from the following aspects. Firstly, we respectively delayed the pilot time by one and two years in each city. Secondly, we excluded the CHARLS data wave 2011. Finally, we changed the data clustering level to province. Table 4 reports the results of robustness tests. The results were consistent with those in time-varying DID model. Therefore, our results were generally robust.

**Table 4:** Robustness test

Variables	LTCI delay 1 year	LTCI delay 2 years	Exclude data wave 2011	Cluster to province
	Depression	Depression	Depression	Depression
Treat*Post	−1.2751***	−2.3461***	−0.6325**	−0.4168*
	(0.2769)	(0.8412)	(0.2930)	(0.2060)
Control variables	Yes	Yes	Yes	Yes
Fixed city	Yes	Yes	Yes	Yes
Fixed year	Yes	Yes	Yes	Yes
_cons	14.4485***	14.3436***	14.2168***	15.3810***
	(0.8514)	(0.8499)	(0.9834)	(0.8713)
N	9279	9279	7649	9279
R^2^	0.118	0.118	0.116	0.091

*Note* *, **, and *** represent significant levels of 10%, 5%, and 1%, respectively. The values in parentheses are city-level clustering robust standard errors

**Fig. 3:**
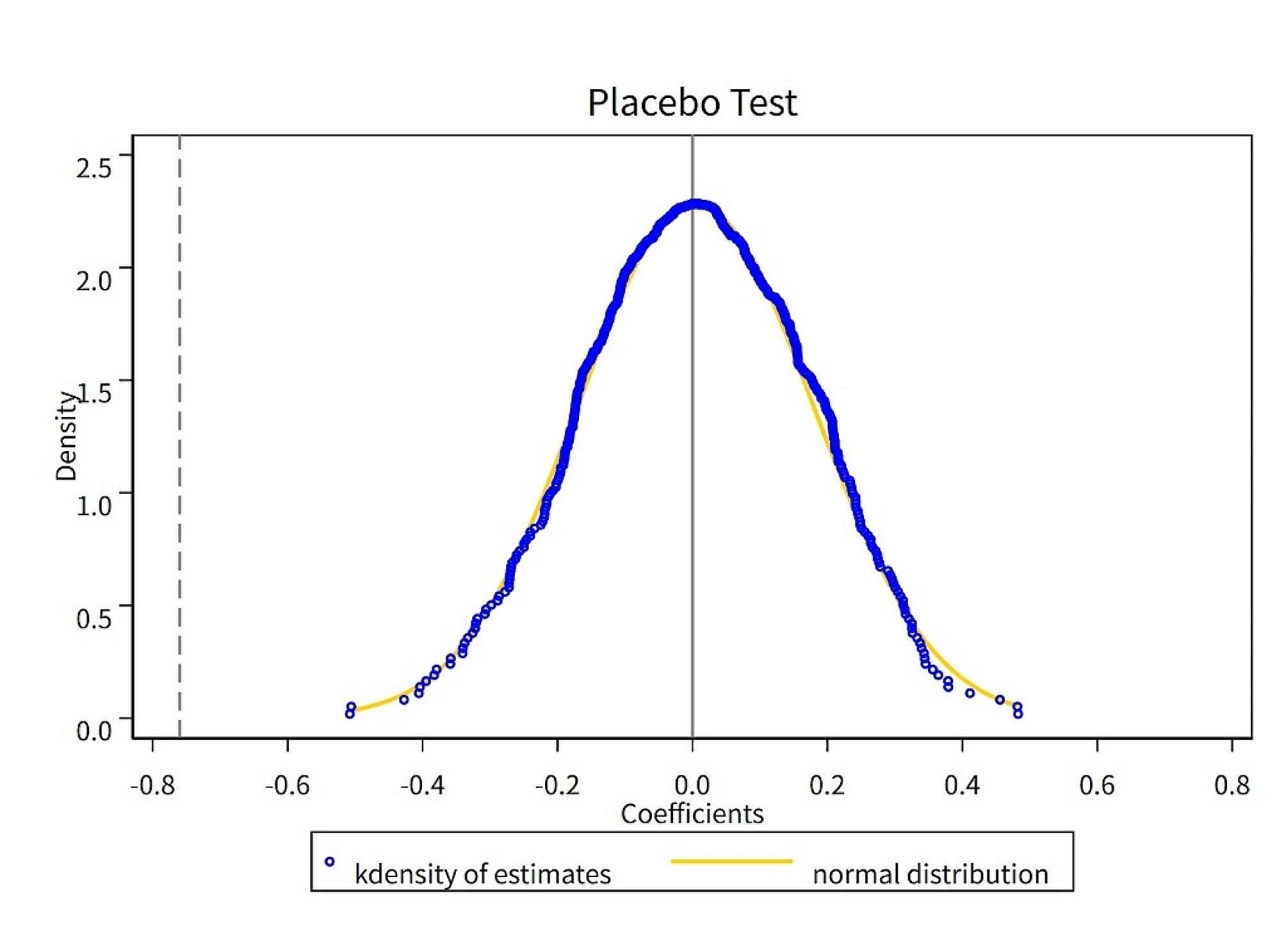
Placebo tests

We also conducted a placebo test by randomly selecting the treated group and pilot time and repeating this random selection process 500 times. The kernel density distribution of the regression coefficients was shown in Fig. 3. It can be found that the regression coefficients were centrally distributed around the 0 value, obeying the normal distribution, while the actual estimated coefficient (vertical dotted line) was obviously an outlier, which indicated that the randomness factor had no statistically significant effect on our research results, the placebo test passed.

### Analysis of heterogeneity

From our main results, LTCI policy statistically significant reduced depression in non-disabled people. Based on the heterogeneity of effects, it is more conducive to improving LTCI policy framework that match health equity in non-disabled people. However, previous LTCI policy effect studies lack in-depth discussion on retirement, financial support, and social participation heterogeneity. Therefore, we explored the reduction effects heterogeneity on retirement, financial support, and social participation (see Table 5).

Table 5 indicates that LTCI policy statistically significant reduced the depression by 0.8267 units (significant level was 5%) in non-disabled unretired people, by 0.7079 units (significant level was 10%) in non-disabled people with financial support and 1.2161 units (significant level was 1%) in non-disabled people without social participation. However, LTCI policy did not statistically significant reduce depression in non-disabled people who were retired, without financial support and with social participation. In other words, the results indicated that non-disabled people who unretired, with financial support and without social participation benefited more from LTCI policy. Thus, our hypotheses 2–4 were validated.

**Table 5:** Retirement, financial support, and social participation heterogeneity of non-disabled people

Variables	Retired	Unretired	With financial support	Without financial support	With social participation	Without social participation
	Depression	Depression	Depression	Depression	Depression	Depression
Treat*Post	−0.0230	−0.8267**	−0.7079*	−0.8012	−0.4531	−1.2161***
	(0.5507)	(0.3435)	(0.3596)	(0.5606)	(0.4086)	(0.3890)
Control variables	Yes	Yes	Yes	Yes	Yes	Yes
Fixed city	Yes	Yes	Yes	Yes	Yes	Yes
Fixed year	Yes	Yes	Yes	Yes	Yes	Yes
_cons	17.5434***	13.3955***	14.3018***	13.9912***	13.7482***	14.5381***
	(2.4940)	(1.0613)	(1.0076)	(1.3761)	(1.1134)	(1.2563)
N	1476	7795	6534	2745	4567	4712
R^2^	0.191	0.108	0.119	0.151	0.136	0.118

*Note* *, **, and *** represent significant levels of 10%, 5%, and 1%, respectively. The values in parentheses are city-level clustering robust standard errors

## Discussion

In this study using nationally representative sample and time-varying DID method, we demonstrated that LTCI policy statistically significant reduced depression in non-disabled people aged 45 years and older in pilot cities compared to non-pilot cities. This result indicated that when LTCI policy was implemented in pilot cities, it affected not only the health of traditional recipients -- disabled people and caregivers, but also affected the health of traditional non-recipients -- non-disabled people. That is, positive health effect spilled over to non-recipients. KAP theory offers a potential explanation for this positive health spillover effect [35–37]. KAP theory asserts that changes in people’s knowledge and attitudes lead to changes in health behaviors, people’s health behaviors follow the logical chain of information-knowledge-attitude-practice-improve health [31–37]. In the LTCI implementing process, whether it was the government’s policy publicity, interpretation and practice, or people’s initiative understand to the policy, the non-disabled people could obtain more knowledge related to aging, disability, and health than before, promote their formation of positive health attitudes and health behaviors, and ultimately lead to the improvement of depression. Taken together, our finding suggested that implementing LTCI policy statistically significant reduced depression in non-disabled people aged 45 years and older, which might partly reveal positive health spillovers of LTCI policy on non-recipients.

There were several potential heterogeneities on depression-reducing effect of LTCI in non-disabled people. Firstly, our results found that non-disabled unretired people benefited more from LTCI policy. Social identity theory offers a potential explanation for this heterogeneity on retirement status. Social identity theory asserts that group member could recognize the emotional and valuable significance that comes with group membership [38]. For adults who have been working for years, retirement means reducing contact or leaving important relationships with co-workers. If these relationships are positive, retirement means a loss of supportive and meaningful interactions, which are associated with worsening health [33]. In other words, retired people are more likely to be depressed than non-retired people, because retirement means the loss of group membership, pronging to an identity crisis, and further causing depression risk among non-disabled people [41, 42]. In addition, non-disabled unretired people could get the depression improvement by gaining more positive health knowledge, attitudes, and health behaviors during the LTCI implementation process. Therefore, healthy knowledge-attitude-practice coupled with positive social identity makes non-disabled unretired people benefit more from LTCI policy.

Secondly, our results suggested that non-disabled people with financial support benefited more from LTCI policy. Social exchange theory offers a potential explanation for this heterogeneity on financial support. Social exchange theory argues that pursuit personal interests is a basic trait of human nature, interaction between people is based on exchange rewards [37]. As people age, the ratio of rewards to costs associated with social interaction might vary based on social status and personal resources. older people’s resources (e.g., health, income, and job roles, etc.) decrease with age, so that older people are more likely to be in unequal social exchange, result in a power disadvantage and basic needs dependence [38, 39]. Financial support provided to older persons can be seen as reimbursement for support received at earlier points in time. Adult children can repay their parents’ financial support during their adult years or during their parents’ old age. According to social exchange theory, parents without financial support are more likely to be depressed than parents with financial support, because financial support given by children to their parents is a manifestation of a good interactive relationship, if the parent-child interaction or reciprocal relationship is not good, their conflict can lead to neglect, abuse, or mistreatment, and further trigger depression in parents [46, 47]. At the same time, the non-disabled people with financial support, through the acquisition of health knowledge and positive thinking during LTCI policy implementation, their attitudes and health behavior will change positively, and eventually lead to depression improvement. Therefore, healthy knowledge-attitude-practice coupled with positive social exchange makes non-disabled people with financial support benefit more from LTCI policy.

Finally, our results suggested that non-disabled people without social participation benefited more from LTCI policy. Activity theory offers a potential explanation for this heterogeneity on social participation. Activity theory believes that the best way to face aging is achieving different social status or social roles by social participation and maintaining the same lifestyle as middle age[ [48]. Social participation might support older adults to maintain life independence and live actively in the community, increase optimism acceptance of change, safe relationships and tolerance in older adults, and thus reduce the depression risk [10]. According to activity theory, people without social participation were more likely to be depressed than people with social participation [49–53]. Although appropriate social participation was beneficial to improve depression, the U-shaped relationship between social participation and depression also indicates that the absence of social participation may not be frightening [10]. Through some channels, such as government multimedia policy advocacy in the LTCI policy process, the absence of social participation did not prevent individuals from gaining more health knowledge, attitudes and behaviors than before. Therefore, healthy knowledge-attitude-practice coupled with the absence of social participation may also make non-disabled people without social participation benefit more from LTCI policy.

Nevertheless, our study also has limitations. First, depression data in CHARLS was self-reported and may be susceptible to memory biases. Second, due to data unavailability, only 15 LTCI pilot cities were included in our study. This limitation should be addressed in future study. Finally, factors that influence depression are abundant, while our study controlled only some of them. Other control factors could be further analyzed in future prospective study.

## Conclusions

In conclusion, our study found that after the implementation of LTCI policy in China, depression in non-disabled people was statistically significant reduced, and this depression-reducing effect of LTCI was heterogeneous among non-disabled people with different retirement, financial support, and social participation condition. Our findings highlight the depression-reducing effect of LTCI policy in non-recipients and suggest that non-disabled people who are unretired, with financial support, or without social participation should not be overlooked during the implement progress of LTCI policy.

## Data Availability

CHARLS data are publicly available at http://charls.pku.edu.cn/en/.
